# Flaxseed oil ameliorated high-fat-diet-induced bone loss in rats by promoting osteoblastic function in rat primary osteoblasts

**DOI:** 10.1186/s12986-019-0393-0

**Published:** 2019-10-17

**Authors:** Fulian Chen, Yan Wang, Hongwei Wang, Zhenhua Dong, Yan Wang, Mengqi Zhang, Jiaxuan Li, Shanshan Shao, Chunxiao Yu, Zhikun Huan, Jin Xu

**Affiliations:** 1 Department of Endocrinology, Shandong Provincial Hospital affiliated to Shandong University, Shandong Provincial Key Laboratory of Endocrinology and Lipid Metabolism, Institute of Endocrinology and Metabolism, Shandong Academy of Clinical Medicine, Jinan, Shandong 250021 People’s Republic of China; 2 Department of Endocrinology, Affiliated Yidu Central Hospital of Weifang Medical College, Weifang, Shandong 262500 People’s Republic of China; 3 Department of Endocrinology, The Second Affiliated Hospital of Shandong First Medical University, Tai’an, Shandong 271000 People’s Republic of China; 4 Department of Endocrinology, People’s Hospital of Rizhao, Rizhao, Shandong 276800 People’s Republic of China; 5 Department of Endocrinology, Jinan Central Hospital affiliated to Shandong University, Jinan, Shandong 250021 People’s Republic of China

**Keywords:** Flaxseed oil, α-Linolenic acid, Osteoporosis, Primary osteoblasts, High-fat diet

## Abstract

**Background:**

α-Linolenic acid (ALA) is a plant-derived omega-3 unsaturated fatty acid that is rich in flaxseed oil (FO). The effect of FO on bone health is controversial. This study aims to evaluate the effect of FO on bone damage induced by a high-fat diet (HFD) and to explore the possible mechanism.

**Methods:**

Male Sprague-Dawley rats were fed a normal control diet (NC, 10% fat), FO diet (NY, 10% fat), HFD (60% fat), or HFD containing 10% FO (HY, 60% fat) for 22 weeks. Micro CT and three-point bending tests were conducted to evaluate bone microstructure and biomechanics. Serum was collected for the detection of ALP, P1NP, and CTX-1. Rat primary osteoblasts (OBs) were treated with different concentrations of ALA with or without palmitic acid (PA) treatment. The ALP activity, osteogenic-related gene and protein expression were measured.

**Results:**

Rats in the HFD group displayed decreased biomechanical properties, such as maximum load, maximum fracture load, ultimate tensile strength, stiffness, energy absorption, and elastic modulus, compared with the NC group (***p*** < 0.05). However, HY attenuated the HFD-induced decreases in bone biomechanical properties, including maximum load, maximum fracture load, and ultimate tensile strength (*p* < 0.05). Trabecular bone markers such as trabecular volume bone mineral density (Tb. vBMD**),** trabecular bone volume/total volume (Tb. BV/TV), trabecular number (Tb. N), trabecular thickness (Tb. Th) were decreased, trabecular separation (Tb. Sp) and the structure model index (SMI) were increased in the HFD group compared with the NC group, and all parameters were remarkably improved in the HY group compared to the HFD group (p < 0.05). However, cortical bone markers such as cortical volume bone mineral density (Ct. vBMD), cortical bone volume/total volume (Ct. BV/TV) and cortical bone thickness (Ct. Th) were not significantly different among all groups. Moreover, the serum bone formation markers ALP and P1NP were higher and the bone resorption marker CTX-1 was lower in the HY group compared with levels in the HFD group. Compared with the NC group, the NY group had no difference in the above indicators. In rat primary OBs, PA treatment significantly decreased ALP activity and osteogenic gene and protein (β-catenin, RUNX2, and osterix) expression, and ALA dose-dependently restored the inhibition induced by PA.

**Conclusions:**

FO might be a potential therapeutic agent for HFD-induced bone loss, most likely by promoting osteogenesis.

## Background

With the development of the economy, saturated fats are generally enriched in modern diets. Diet is one of the risk factors for osteoporosis in addition to heredity, age, oestrogen deficiency, and calcium and phosphorus metabolism [1]. Several studies have indicated that a high-fat diet (HFD) had a negative impact on bone health [2]. One middle-to-older-aged population study indicated that a high body fat percentage was a risk factor for osteoporosis with ageing [3]**.** Two animal studies have reported that HFD consumption was associated with bone strength reduction and that adverse microstructure changes occurred in the cancellous bone compartment [4, 5]. In addition, Ahmad Alsahli et al. noted that elevated levels of palmitic acid (PA) reduced osteoblast (OB) function in vitro and bone formation markers in vivo [6].

As diet is modifiable, the study of nutrition on bone health may provide an approach for osteoporosis prevention. Omega-3 unsaturated fatty acids (ω-3 FA) included eicosapentaenoic acid (EPA), docosahexaenoic acid (DHA) and a-linolenic acid (ALA), which can lower lipid disorders, reduce inflammatory factors, resist arteriosclerosis [7, 8]. Recent studies have shown that ω-3 FA has protective effects on bone metabolism [9–11]. Studies have shown that bone loss induced by aging, oestrogen deficiency, ovariectomized (OVX) animals and inflammation can be abrogated by DHA and EPA [12–14]. However, the effect of ALA on bone health is controversial. Flaxseed oil (FO) contains approximately 60% ALA and represents a plant-derived ω-3 FA. A clinical study indicated that daily consumption of 6 g/d of FO may reduce bone resorption in haemodialysis patients [15]. An animal study showed that ALA associated with calcium and protein provided by flaxseed flour contributed to bone quality in the post-partum period of dam rats [16]. Longo et al. suggested that providing FO, possibly through its high ALA content, provided protection against OVX-induced alveolar bone loss in rats [17]**.** However, Cohen et al. noted that feeding a 10% FO diet did not have effects on bone mass and bone strength compared with a 10% corn oil diet in growing mice [18]. However, another animal study showed that lifelong intake of FO or menhaden oil modulated bone microarchitecture during growth but did not affect OVX-induced bone loss in Sprague-Dawley (SD) rats [19]**.** Considering these contradictory reports in the scientific literature, the effectiveness of FO is not completely proven.

Whether FO has a protective effect on HFD-induced bone damage has not been clarified, and the specific mechanism of bone protection is unclear. Therefore, this study aimed to evaluate whether totally replacing soybean oil and partly replacing lard with FO in HFD can improve high-fat-induced bone damage in vivo. We treated rat primary OBs with ALA in vitro to observe the effect of ALA on osteoblastic function, osteogenic-related gene and protein expression and tried to explore the mechanism of ALA on bone protection.

## Material and methods

### Animal experiments

Forty 4-week-old male SD rats weighing 100–120 g were obtained from Vital River Laboratory Animal Technology Co Ltd. (Beijing, China). Animals were housed 2 per cage, maintained in constant temperature-controlled rooms (22–25 °C) with a 12-h light/dark cycle, and had free access to food and water. After being acclimated to the housing conditions for 1 week, they were randomly assigned to one of the four diets: (1) (NC group, *n* = 10): normal control diet, fat provides 10% of energy; (2) (NY group, n = 10): FO substitutes for all of the soybean oil and lard in the normal control diet, fat provides 10% of energy; (3) (HFD group, n = 10): fat provides 60% of energy; and (4) (HY group, n = 10): FO replaces all of the soybean oil and part of the lard in the HFD, fat provides 60% of energy. The rats were fed the specified diet for 22 weeks before they were sacrificed. The composition of the experimental diets was assayed by the Beijing Research Institute for Nutritional Resources, and details are shown in Table 1. To avoid potential oxidation, each diet was repackaged into weekly portions, vacuum-sealed, and stored at 4 °C in the dark until use.

**Table 1:** Composition of experimental diets (4057 kcal per serving)

Raw material Configuration(g)	NC (D12450B)	NY	HFD (D12492)	HY
Casein lactic	200	200	200	200
L-Cystine	3	3	3	3
Corn Starch	315	315	0	0
Maltodextrin	35	35	125	125
Sucrose	350	350	68.8	68.8
Cellulose BW	50	50	50	50
Soybean Oil	25	0	25	0
Flaxseed Oil	0	45	0	45
Lard	20	0	245	225
Mineral Mix S10026	10	10	10	10
DiCalcium Phosphate	13	13	13	13
Calcium Carbonate	5.5	5.5	5.5	5.5
Potassium Citrate	16.5	16.5	16.5	16.5
Vitamin Mix V10001	10	10	10	10
Choline Bitartrate	2	2	2	2
Summation(g)	1055	1055	773.8	773.8
Energy (kcal)	4057	4057	4057	4057

### Serum sample and bone tissues acquisition

All rats were fasted for 12 h and anaesthetized with 1% pentobarbital sodium after feeding the specified diet for 22 weeks. Serum was collected after centrifuging at 3000 rpm for 10 min and stored at *−*80 °C for subsequent analyses. After removing the soft tissue, the right femurs and left tibias were removed and stored in 4% paraformaldehyde for micro-computed tomography (microCT) analysis and bone histology analysis. Left femurs were wrapped in saline gauze and frozen at −30 °C for the three-point bending test.

### Bone biomechanical analysis

To assess bone strength, the three-point bending test was performed on a universal testing machine (BoseElectroForce®3230, Bose Corporation, USA) by placing the bones, anterior face up, on two supports that were equidistant from the ends and 16 mm apart. The load was applied at a constant deformation rate of 2 mm/min. The diaphysis of the femur was loaded until a fracture occurred to determine the yield and fracture parameters. The yield represents the point at which bone ceases to behave elastically. The data were automatically recorded in a computer interfaced to the testing machine, and a typical load-deformation curve was created. A saline solution was used to keep specimens moist during testing. The material properties of the bones were calculated as described in previous studies [20]. The maximum load, maximum fracture load, energy absorption, stiffness, ultimate tensile strength and elastic modulus were measured.

### Bone microCT analysis

The trabecular and cortical microarchitectures of the right femurs were scanned using microCT (SkyScan-1176 μCT, Brook Corporation, Belgium), and the images were reconstructed to an isotropic voxel size of 12 μm. The volume of interest (VOI), which was located 1 mm from the metaphyseal line to the 100 continuous slices above, was selected for data analysis. All 3D image manipulations and analyses were performed using system software (MicroView, v.2.1, GE Healthcare).

### Bone histology analysis

Left tibias were fixed in 4% paraformaldehyde (Sigma*-*Aldrich), decalcified in 10% ethylenediaminetetraacetic acid (Sigma-Aldrich) at pH 7.0, and then embedded in paraffin. Longitudinal sections (5-μm thick) were stained with haematoxylin and eosin to observe the number of trabecular and bone marrow adipocytes. All pathological images were observed using a light microscope from several visual fields per slice (Axiovert 100 M Zeiss, Zeppelinstrasse, Germany) at 100× magnification. The number of trabecular and adipocyte analyses was determined by a pathologist who was blind to the grouping situation according to the results of the images.

### Biochemical analysis

Serum levels of alkaline phosphatase (ALP) were measured using an Olympus AU5400 automatic biochemical analyser (Olympus Co, Ltd., Tokyo, Japan). Serum levels of procollagen type 1 N-terminal propeptide (P1NP) were measured using an ELISA kit (Cusabio, Wuhan, China). C-Telopeptide of type 1 collagen (CTX-1) was also measured using an ELISA kit (Cusabio, Wuhan, China). All measurements were conducted according to the manufacturer’s instructions.

### Primay OBs isolation and cell culture

Primary OBs were isolated from the skulls of newly born (72 h) SD rats. The standardized extraction procedure of primary OBs was carried as previously described [21, 22]. The cells were collected and dissolved in DME/F-12 1:1 culture medium (HyClone) supplemented with 10% foetal bovine serum (Excel), penicillin-streptomycin mixed liquor (Solarbio) and incubated in a humidified atmosphere of 95% air and 5% CO2 at 37 °C. The third-generation OBs were used in follow-up studies.

### ALP staining assay

Primary OBs were cultured in 24-well plates at a density of 1*10^4^ cells per well. After an initial 24 h period in growth medium (time 0), cells were treated with vehicle alone, ALA (Sigma) (1 μM, 10 μM, or 100 μM) or PA (Sigma) 0.2 mM or (PA 0.2 mM + ALA 1 μM) or (PA 0.2 mM + ALA 10 μM) or (PA 0.2 mM + ALA 100 μM). On the fifth day of culture, cells were stained with the ALP reagent kit (Genmed, Shanghai, China) according to the manufacturer’s protocol. The absorbance was measured at a wavelength of 520 nm according to the manufacturer’s protocol.

### Real-time quantitative PCR (RT-qPCR)

Primary OBs were treated with vehicle alone, ALA (1 μM, 10 μM, or 100 μM) or PA 0.2 mM or (PA 0.2 mM + ALA 1 μM) or (PA 0.2 mM + ALA 10 μM) or (PA 0.2 mM + ALA 100 μM) for 3 days. Total RNA was extracted from OBs using TRIzol (Takara Biotech, Dalian, China) according to the manufacturer’s protocol. cDNA was obtained from 1 μg of total RNA, and RT-qPCR was carried out using the SYBR Green PCR Master Mix reagent kit (Takara Biotech, Dalian, China). The primer sequences used are listed in Table 2. PCR products were subjected to a melting curve analysis, and the relative expression was calculated for each gene by the 2^-∆∆CT^ method, using β-actin for normalization. *β-Catenin, RUNX2, and osterix* mRNA (Takara Biotech, Dalian, China) expression levels were examined on day 3 after different treatments. Each sample was measured at least in triplicate.

**Table 2:** Primer sequences used for the determination of gene expression

Gene (Rats)	Primer sequence((5′ - 3′)	Product bp
β-catenin	Forward CATCACCACGCTGCATAATC	156
	Reverse GAGCTTGCTTTCCTGATTGC	
RUNX2	Forward CATGGCCGGGAATGATGAG	148
	Reverse TGTGAAGACCGTTATGGTCAAAGTG	
os0074erix	Forward CACCCATTGCCAGTAATCTTCGT	97
	Reverse GGACTGGAGCCATAGTGAGCTTCT	
β-actin	Forward ACCCAGATCATGTTTGAGAC	99
	Reverse GTCAGGATCTTCATGAGGTAGT	

β-catenin, catenin beta1; RUNX2, runt-related transcription factor 2; osterix, Sp7 transcription factor; β-actin, actin, beta

### Western blot analysis

Primary OBs were treated with vehicle alone, ALA (1 μM, 10 μM, or 100 μM) or PA 0.2 mM or (PA 0.2 mM + ALA 1 μM) or (PA 0.2 mM + ALA 10 μM) or (PA 0.2 mM + ALA 100 μM) for 3 days. OBs were scraped from the culture dish and washed twice with ice-cold PBS. Total protein was extracted from the cell pellet using RIPA lysis buffer (Shenergy Biocolor Bioscience & Technology Co, Shanghai, China) according to the manufacturer’s protocol. Protein concentrations were quantified using a BCA protein quantitative assay kit (Shenergy Biocolor Bioscience & Technology Co, Shanghai, China). The total proteins were separated by 10% SDS-PAGE and transferred to a 0.45-μm PVDF membrane (Millipore, Billerica, MA, USA). The membrane was incubated at room temperature in a blocking solution of 5% skimmed milk powder dissolved in TBST containing 0.05% Tween for 1 h, followed by incubation with the primary antibodies overnight at 4 °C. The membrane was washed three times in TBST (10 min each wash) and incubated with horseradish peroxidase-conjugated anti-rabbit (Abcam, ab136817) or anti-mouse (Abcam, ab136815) IgG secondary antibody at a 1:5000 dilution in the blocking solution. The blots were exposed to enhanced chemiluminescence (Pierce, Rockford, IL, USA). The antibodies used in this study included mouse GAPDH (glyceraldehyde-3-phosphate dehydrogenase) antibody at a 1:1000 dilution (Sigma-Aldrich A5441), rabbit β-catenin antibody at a 1:1000 dilution (Cell Signaling Technology, #9562), mouse RUNX2 antibody at a 1:500 dilution (Cell Signaling Technology,8486 s), and rabbit osterix antibody at a 1:500 dilution (Abcam, ab209484). The relative target protein levels were normalized to GAPDH in the same membrane.

### Statistical analysis

Data were analysed by SPSS 18.0 software and were expressed as the mean ± standard deviation (SD). Means were compared using unpaired Student’s *t-*tests for comparisons between two groups and one-way ANOVA (Sidak’s multiple comparisons test) for comparisons among multiple groups. A two-tailed *P* value < 0.05 was considered significant.

## Results

### FO improved bone biomechanical properties in HFD-induced bone damage

**Fig. 1:**
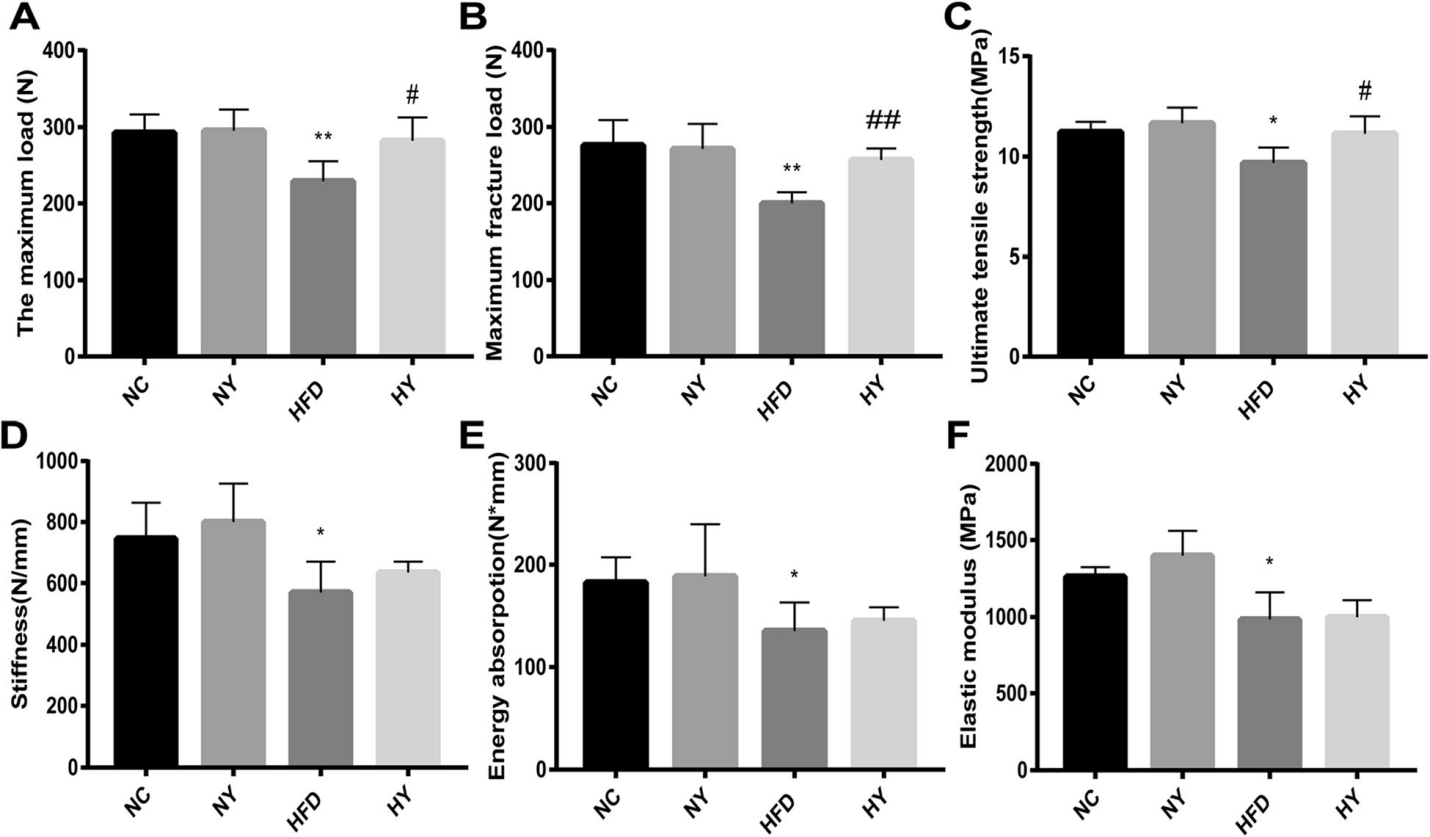
The effect of FO on bone biomechanical properties. (**a**) The maximum load of the bone before crushing. (**b**) Maximum fracture load of the bone crushing. (**c**) Ultimate tensile strength of the bone before brittle fracture. (**d**) Stiffness, the slope of the linear region. (**e**) Energy absorption, AUC of the load multiplied by the displacement. (**f**) Elastic modulus, maximum slope of the stress-strain curve. Data represent the mean ± SD (*n* = 8). **P < 0.01, *P < 0.05 versus the NC group and ## P < 0.01, # *p* < 0.05 versus the HFD group by one-way ANOVA

The results of the three-point bending test in the femur samples showed that the biomechanical properties, such as the maximum load (*p* = 0.0012), maximum fracture load (*p* = 0.0004), ultimate tensile strength (*p* = 0.0242), stiffness (*p* = 0.0138), energy absorption (*p* = 0.0429), and elastic modulus (*p* = 0.0112), were decreased in the HFD group compared with the NC group after four different diets for 22 weeks. The maximum load (*p* = 0.0467), maximum fracture load (*p* = 0.0192), and ultimate tensile strength (*p* = 0.0264) were significantly enhanced in the HY group compared with the HFD group, but the stiffness, energy absorption, and elastic modulus showed no significant differences between the HY group and the HFD group. Moreover, compared with the NC group, the NY group had no difference in the above indicators (Fig. 1a-f).

### FO ameliorated trabecular bone damage in HFD-induced bone loss

**Fig. 2:**
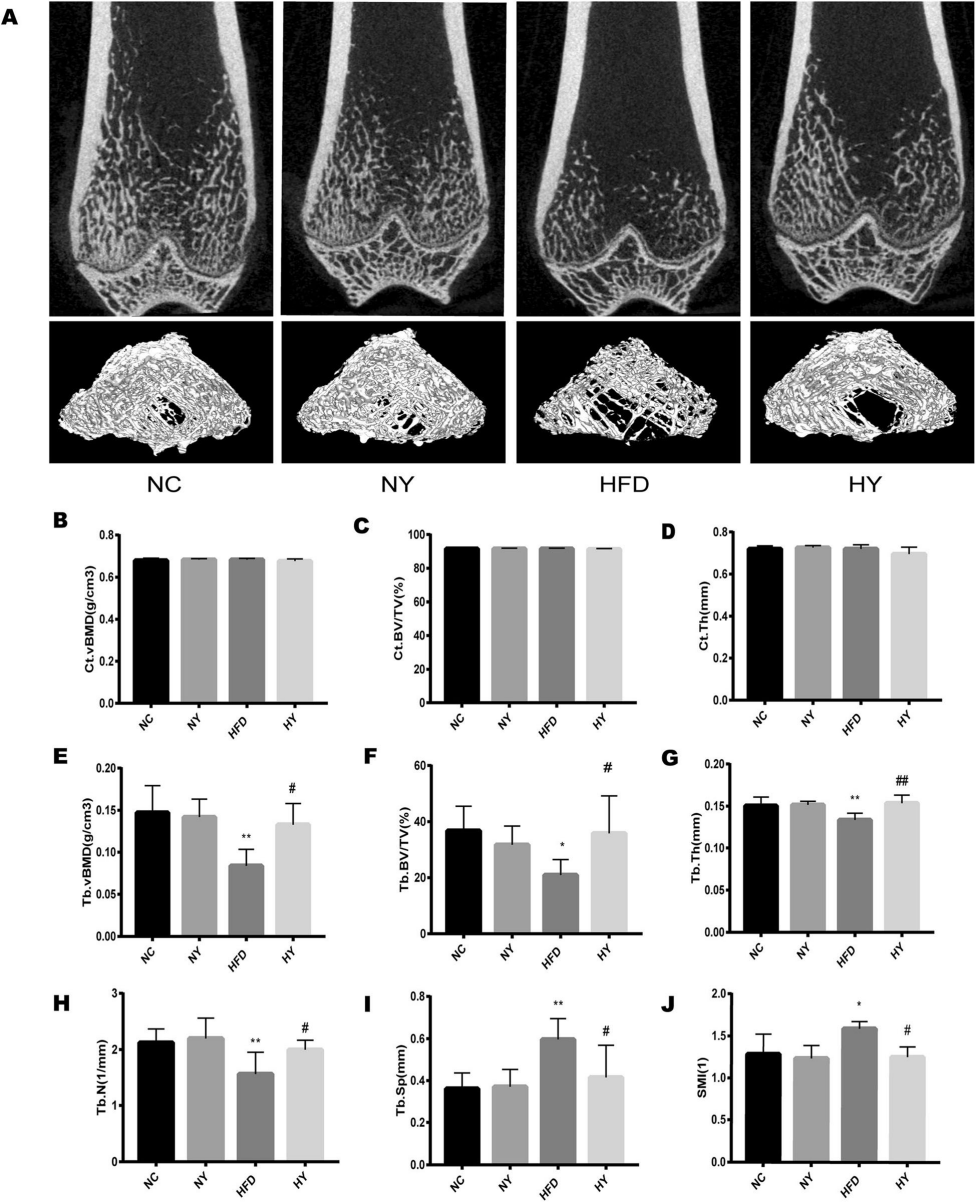
The effect of FO on bone structural characteristics by microCT. (**a**) Representative 2D and 3D images of microCT reconstruction of distal femurs. Scale bar, 500 μm. (**b-d**) MicroCT analysis of cortical bone parameters: Ct. vBMD, cortical volumetric bone mineral density; Ct. BV/TV, cortical bone volume/total volume; Ct. Th, cortical bone thickness. (**e-J**) MicroCT analysis of trabecular bone parameters: Tb. vBMD, trabecular volumetric bone mineral density; Tb. BV/TV, trabecular bone volume/total volume; Tb. Th, trabecular thickness; Tb. N, trabecular number; Tb. Sp, trabecular separation; SMI, structure model index. The results are shown as the mean ± SD (*n* = 6). **P < 0.01, *P < 0.05 versus the NC group and ## *P* < 0.01, # *p* < 0.05 versus the HFD group by one-way ANOVA

Consistent with the results of the three-point bending test, the trabecular volume bone mineral density (Tb. vBMD) (*p* = 0.0011), trabecular bone volume/total volume (Tb. BV/TV) (*p* = 0.0161), trabecular number (Tb. N) (*p* = 0.0042), and trabecular thickness (Tb. Th) (*p* = 0.0087) were decreased and the trabecular separation (Tb. Sp) (*p* = 0.0009) and structure model index (SMI) (*p* = 0.0348) were increased in the HFD group compared with the NC group after four different diet treatments for 22 weeks, and all of the above parameters (Tb. vBMD, *p* = 0.0141) (Tb. BV/TV, *p* = 0.0468) (Tb. N, *p* = 0.0288) (Tb. Th, *p* = 0.0035) (Tb. Sp, *p* = 0.0180) (SMI, *p* = 0.0182) were markedly improved in the rats of the HY group compared to the HFD group. However, compared with the NC group, the NY group had no difference in the above indicators (Fig. 2a, e-j). Meanwhile, the cortical volume bone mineral density (Ct. vBMD), cortical bone volume/total volume (Ct. BV/TV) and cortical bone thickness (Ct. Th) had no significant differences among the four groups (Fig. 2a-d).

### FO changed the trabecular number and the number of adipocytes

**Fig. 3:**
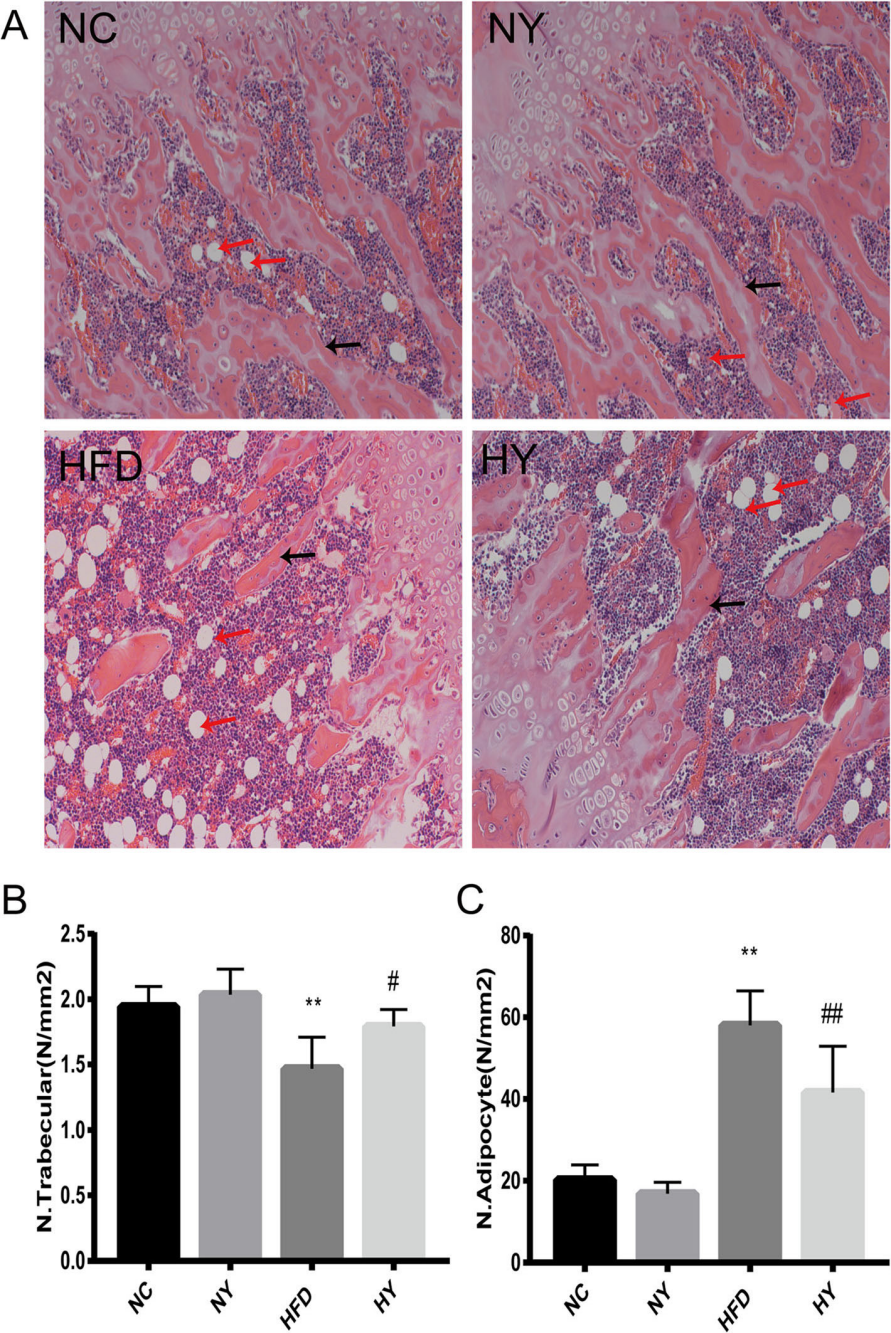
H&E staining to observe the trabecular number and number of adipocytes from rat tibial sections. (**a**) Representative images are shown. Scale bar, 100 μm. The black arrows indicate the trabecular bone structure. The red arrows represent adipocytes. (**b**) The trabecular number (N. trabecular) per square millimetre was evaluated (*n* = 5). (**c**) The number of adipocytes (N. adipocytes) per square millimetre was evaluated (n = 5). **P < 0.01, *P < 0.05 versus the NC group and ## P < 0.01, # *p* < 0.05 versus the HFD group by one-way ANOVA

The results of HE staining in the right tibia samples showed that the trabecular number was decreased in the HFD group compared with the NC group (*p* = 0.0027), while the HY group markedly increased the trabecular number compared with the HFD group (*p* = 0.0408) (Fig. 3a-b). We also found that the number of adipocytes in the HFD group was increased compared with the NC group (*p* < 0.0001), while it was dramatically decreased in the HY group compared with the HFD group (*p* = 0.0093) (Fig. 3a and c). Similarly, there were no significant differences between the NC and NY groups (Fig. 3a-c).

### FO increased bone formation markers and decreased bone resorption markers in the HY group compared with the HFD group

**Fig. 4:**
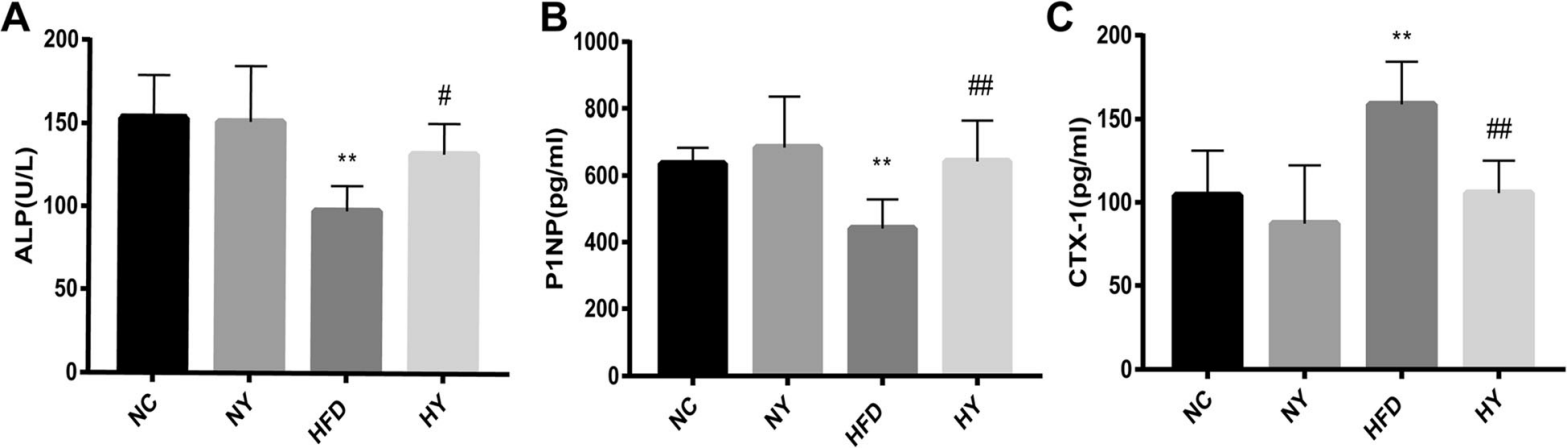
The effect of FO on bone formation markers and bone resorption marker. (**a-b**) bone formation markers: ALP, alkaline phosphatase; P1NP, procollagen type I N-terminal propeptide. (**c**) bone resorption marker: CTX-1, C-telopeptide of type 1 collagen. Data are expressed as the mean ± SD (n = 8). **P < 0.01, *P < 0.05 versus the NC group and ## P < 0.01, # p < 0.05 versus the HFD group by one-way ANOVA

In contrast, the serum levels of the bone formation markers ALP and P1NP were lower in the HFD group than those in the NC group (*p* = 0.0006, 0.0039) and significantly higher in the HY group compared with those in the HFD group (*p* = 0.0426, 0.0057) (Fig. 4a-b). In contrast, the serum levels of CTX-1, a marker for bone resorption, were elevated in the HFD group compared with levels in the NC group (*p* = 0.0023) and were significantly reduced in the HY group compared with those in the HFD group (*p* = 0.0028) (Fig. 4c). However, compared with the NC group, the NY group had no difference in the above indicators (Fig. 4a-c).

### ALA dose-dependently restored the inhibition of ALP activity induced by PA

**Fig. 5:**
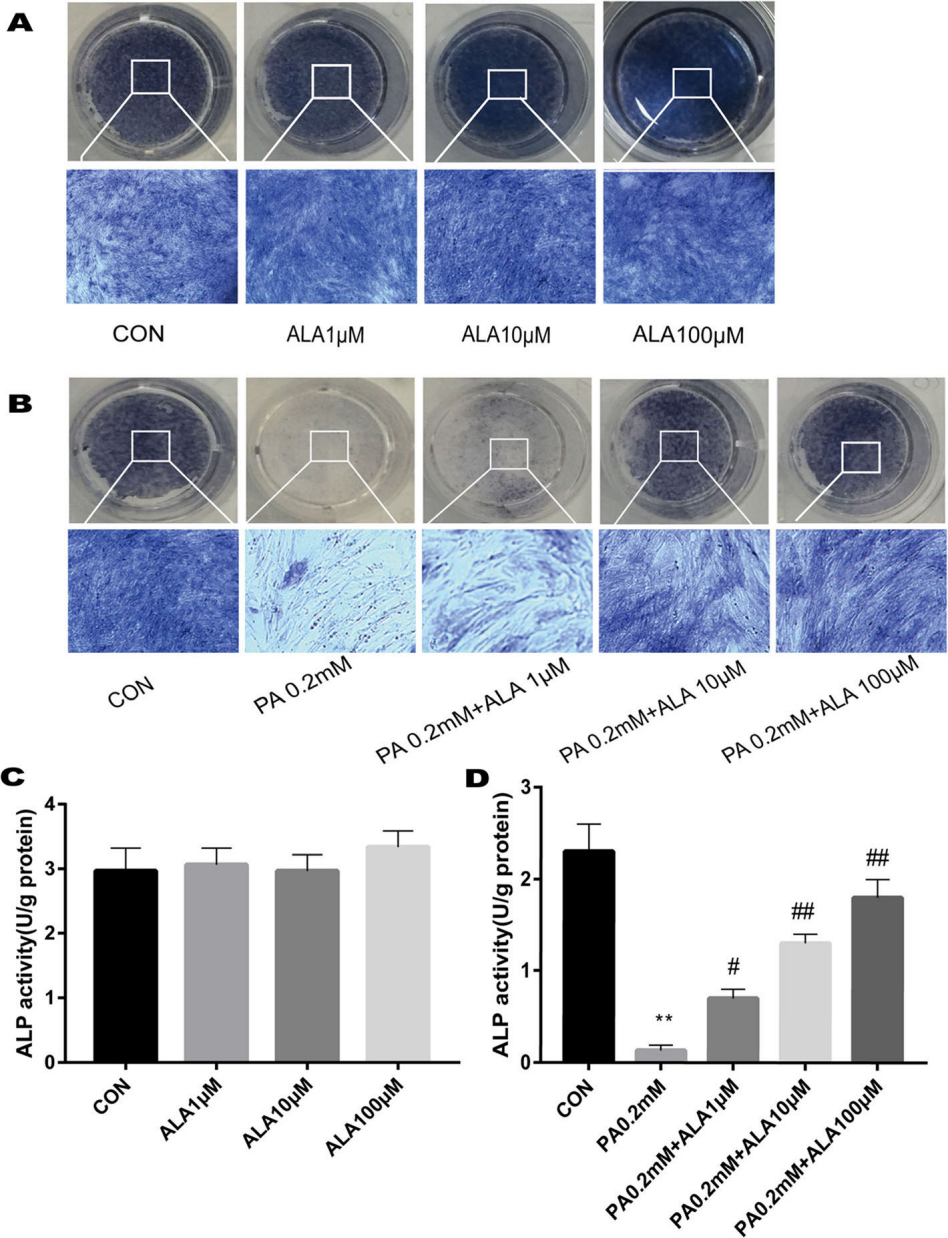
ALP staining in primary OBs. (**a**) OBs were grown for 5 days in the indicated medium (con group contained 0.1% ethanol, ALA 1 μM, ALA 10 μM, ALA 100 μM). (**b**) OBs were grown for 5 days in the indicated medium (con group contained 0.1% ethanol and 2.4% BSA, PA 0.2 mM, PA 0.2 mM + ALA 1 μM, PA 0.2 mM + ALA 10 μM, PA 0.2 mM + ALA 100 μM). (**c-d**) ALP activity quantification was measured after different treatments. Data are shown as the mean ± SD (*n* = 3). ^**^p < 0.01 versus vehicle treatment (con). ## *p* < 0.01 # *p* < 0.05 versus (PA 0.2 mM) treatment by one-way ANOVA

The effects of ALA on ALP activity were also assessed. As shown in Fig. 5a and c, the alteration of ALP activity was not observed in cells with ALA compared with the vehicle control group after 5 days of incubation with different concentrations of ALA. However, ALP activity was significantly decreased after 5 days of PA treatment (0.2 mM) compared with the vehicle control group (*p* < 0.0001), and ALA dose-dependently restored the inhibition of ALP activity induced by PA (PA0.2 mM + ALA1 μM versus PA 0.2 mM, *p* = 0.0177; PA0.2 mM + ALA10 μM versus PA 0.2 mM, p < 0.0001;PA0.2 mM + ALA100 μM versus PA 0.2 mM, p < 0.0001) (Fig. 5b and d).

### ALA promoted osteogenic gene (β-catenin, RUNX2, and osterix) expression and dose-dependently restored the inhibition of PA

**Fig. 6:**
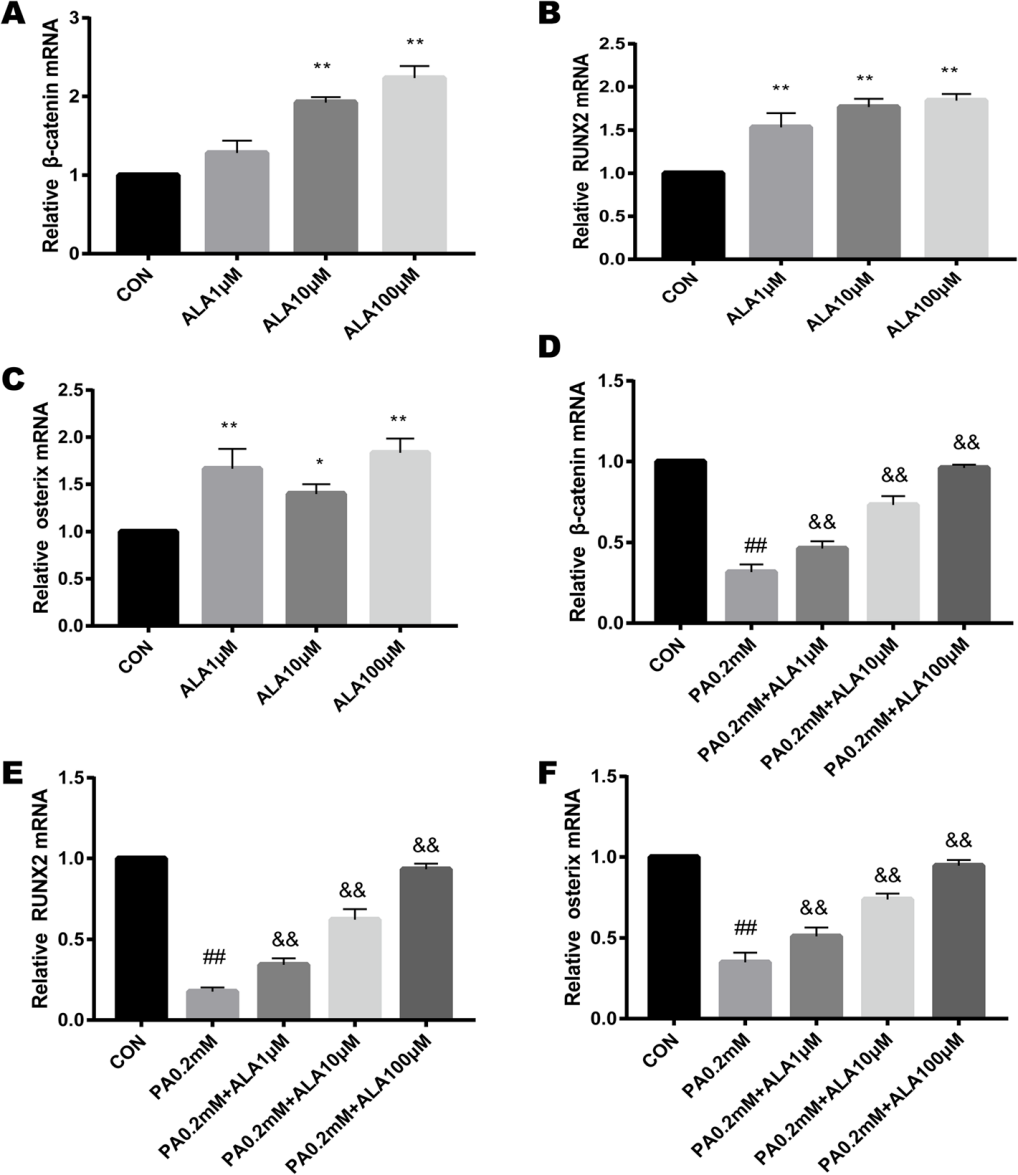
The effect of ALA on osteogenic-related gene expression. RT-qPCR of β-catenin (**a, d**), Runx2 (**b, e**) and osterix (**c, f**) expression in primary OBs subjected to different treatments. Data are expressed as the mean ± SD from three independent experiments. ** *p* < 0.01, * *p* < 0.05 versus the vehicle treatment (con) in Fig. 6a-c. ## *p* < 0.01, # *p* < 0.05 versus the vehicle treatment (con) in Fig. 6d-f, && p < 0.01 & p < 0.05 versus PA 0.2 mM treatment in Fig. 6d-f by one-way ANOVA

To determine whether ALA could promote osteogenic-related gene expression, osteogenic-specific genes, including β-catenin, RUNX2, and osterix were examined in primary OBs. RT-qPCR analysis revealed that OBs treated with ALA showed higher gene expression with the vehicle group (Fig. 6a-c). Meanwhile, we observed the expression of osteogenic genes after PA treatment for 3 days. Consistent with ALP staining, significant decreases in osteogenic relative gene expression were observed (all *p* < 0.0001), and ALA can mitigate the decreases in osteogenic relative gene expression induced by PA in a concentration-dependent manner (all *p* < 0.01) (Fig. 6d-f).

### ALA promoted osteogenic protein (β-catenin, RUNX2, and osterix) expression and dose-dependently restored the inhibition of protein expression induced by PA

**Fig. 7:**
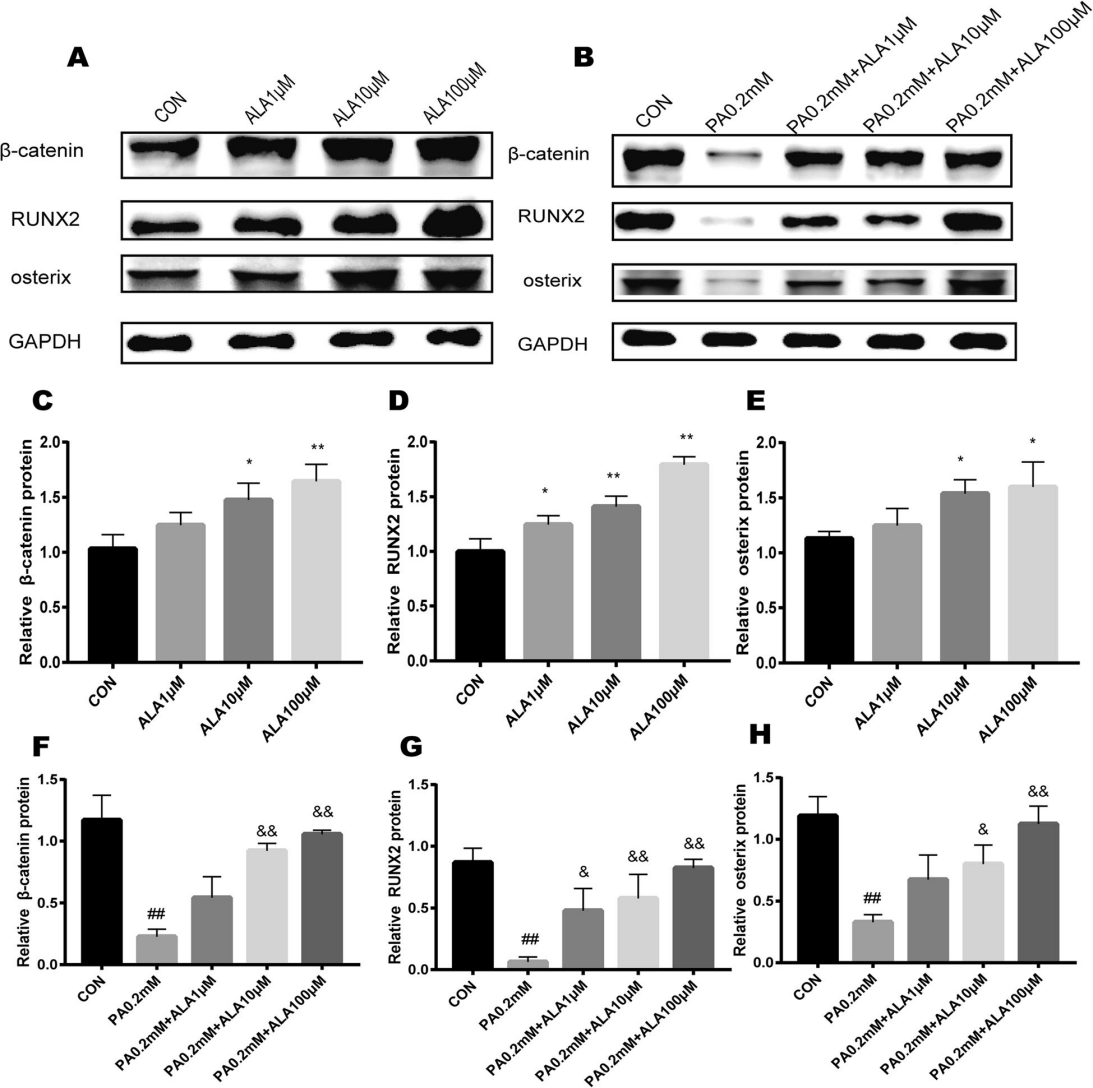
The effect of ALA on osteogenic-related protein expression. (**a-b**) Western blot bands of β-catenin, RUNX2, osterix and GAPDH proteins in primary OBs subjected to different treatments. (**c-h**) Western blotting analysis of β-catenin, RUNX2 and osterix protein. Values were quantified by densitometry and normalized to GAPDH. Data are expressed as the mean ± SD from three independent experiments. ** *p* < 0.01, * *p* < 0.05 versus the vehicle treatment (con) in Fig. 7c-e. ## *p* < 0.01, # *p* < 0.05 versus the vehicle treatment (con) in Fig. 7f-h, && *p* < 0.01, & *p* < 0.05 versus PA 0.2 mM treatment in Fig. 7f-h by one-way ANOVA

Osteogenic-specific proteins, including β-catenin, RUNX2, and osterix, were also examined. Consistent with the RT-qPCR results, western blot analysis revealed that ALA upregulated osteogenic protein expression compared with the vehicle group (Fig. 7a and c-e). β-Catenin, RUNX2, and osterix were significantly decreased after 3 days of PA treatment (all p < 0.0001), and ALA dose-dependently restored the inhibition of osteogenic-specific proteins induced by PA (Fig. 7b and f-h).

## Discussion

The major finding of this study was that FO alleviated bone loss in HFD rats. Meanwhile, ALA can promote primary osteoblastic function through increasing β-catenin/RUNX2/osterix genes and proteins expression and restoring PA induced ALP activity decreases.

Generally, osteoporosis had been considered to be a female disease. However, a recent study indicated that abdominal fat was also an osteoporosis alarm for men [23]**.** Another study indicated that males were more sensitive to diet and benefited from a healthy diet [24]. Obesity induced by HFD aggravated bone loss in the cancellous bone compartment, with a greater loss in males than females **[**25**]**. Our current study also focused on male rats, and trabecular bone loss was observed in HFD induced male SD rats.

The bone loss induced by HFD was attenuated by treatment with FO, as shown by micro-CT**,** three-point bending test and bone histology analysis.

Lipid metabolic disorder has been demonstrated to be detrimental to various organs and was associated with many diseases, such as type 2 diabetes, obesity [26],non-alcoholic fatty liver disease, cardiomyopathy [27], chronic kidney disease [28] and endothelial cell dysfunction [29]. Recent studies have indicated that overnutrition-induced metabolic disturbance could be a possible pathogenic factor in bone metabolism dysfunction, revealing the bone to be another victim of “lipotoxicity”. Cao et al. found that HFD decreased cancellous bone mass but had no effect on cortical bone mass in the tibia in mice [30], which was consistent with our study. Our unpublished data indicated that bone mineral density (BMD) was not significantly different among the four groups after four different diet treatments for 22 weeks by dual-energy X-ray. Combined with the microCT results, we inferred that the effect of HFD on trabecular bone was earlier than that on cortical bone.

Jason A. Inzana et al. showed that immature mice were more susceptible to the detrimental effects of HFD on cancellous bone in the distal femur, which increased the evidence of excess dietary fat as a possible pathologic factor of bone damage [31]. In our study, we observed SD rats starting from week 4 to week 27 and given different dietary interventions for 22 weeks, which was equivalent to the critical period of bone metabolism changes from early childhood to adulthood in humans [32]**.** Consistent with previous reported studies, we discovered that HFD reduced cancellous bone function in male growth-stage SD rats. However, there were no differences, including serological markers, bone biomechanics, microCT structure and bone histology, between the NC group and the NY group. The reason for this is that bone metabolism is in equilibrium when consuming a normal diet, but ALA only works when bone metabolism is imbalanced.

Bone metabolism depends on the dynamic balance between the osteogenesis of OBs and bone resorption by osteoclasts (OCs). At present, research on the bone protection of ALA is uncertain. Most studies indicated that the protective effect of ALA on bone metabolism was through inhibition of OCs [33, 34]**,** and there were no studies on primary OBs. Recent studies indicated that the canonical Wnt/β-Catenin pathway affected BMSC osteogenic differentiation [35]. Wnt signalling can promote osteoblastic precursor differentiation into more differentiated OBs and can serve as a negative regulator of adipogenesis [36]**.** This differentiation process was tightly regulated by complex signalling events. β-Catenin is a key signalling molecule that promotes cell differentiation [37]**.** While many signals were convergent in OBs, the reduction of β-catenin led to osteopenia [38]. Our results showed that ALA dramatically promoted β-catenin gene and protein expression in OBs, which suggested that ALA may promote osteogenic differentiation through the Wnt pathway.

Osteogenesis generally involves three major phases: proliferation, extracellular matrix formation and mineralization, which are regulated by a diverse set of key factors, such as transcription factors, growth factors, hormones and signalling pathways [39]. RUNX2 was known as a specific regulator in osteoblastic differentiation at the whole stage, thus activating the expression of osteogenic-related genes [40]. Furthermore, recent studies have indicated that RUNX2 functions more as a promoter than as a transcription factor, acting as a transcriptional activator or repressor [41]. Osterix is another transcription factor for osteoblastic differentiation and bone formation. Osterix plays an important role in osteoblastic differentiation following RUNX2-mediated mesenchymal condensation [42]. Our study showed that ALA promoted the expression of the osteogenic transcription factors RUNX2 and osterix. PA inhibited the expression of RUNX2 and osterix, and ALA reversed the inhibitory effect of PA in a concentration-dependent manner. However, Casado-Diaz et al. showed that unsaturated fatty acids cannot reverse the inhibitory effect of omega-6 on osteogenesis [43]. The above study was inconsistent with the results of this study because of the different types of cells, intervention time, and application of omega-3 types.

ALP activity reflected the early function of OBs. Treating primary OBs using ALA alone did not change ALP activity significantly. However, when PA and ALA were simultaneously used to stimulate OBs, ALA restored the inhibition induced by PA in a concentration-dependent manner. Similarly, cell experiments also confirmed that under normal conditions, in OBs treated with ALA, only osteogenic gene and protein expression levels were increased, while no ALP activity change was observed. ALA affected the early function of OBs only when OBs were in a state of inhibition.

There were three limitations in this study. One limitation was that the present study was conducted in a rodent model and not in humans. Another limitation was that the mechanism of ALA promoting osteogenesis was explored only from the cellular side, and no in vivo experiments were conducted to investigate the mechanism. The third limitation was that two rats were placed in one cage, and diet intake was not able to be measured. Additionally, the body weight changes were not measured, although the diet intake and body weight changes might be important factors related to bone health. In the next step, we will conduct in vivo experiments to further elucidate the protective mechanism of ALA in alleviating bone damage induced by HFD and provide a theoretical basis for the clinical treatment of bone damage caused by HFD.

## Conclusions

In the present study, our main finding was that FO alleviated bone loss in HFD rats, probably by promoting osteoblastic β-catenin/RUNX2/osterix gene and protein expression and restoring PA-induced ALP activity decreases. These findings indicated that FO might be a potential therapeutic agent for HFD-induced bone loss, most likely by promoting osteogenesis.

## Data Availability

All data generated or analysed during this study are included in this published article or are available from the corresponding author on reasonable request.

## Abbreviations

ALA: α-linolenic acid
ALP: Alkaline phosphatase
CTX-1: C-telopeptide of type 1 collagen
DHA: Docosahexaenoic acid
EPA: Eicosapentaenoic acid
FO: Flaxseed oil
GAPDH: Glyceraldehyde-3-phosphate dehydrogenase
HFD: High-fat diet
HY: High-fat diet containing 10% flaxseed oil
NC: Normal control diet
NY: Flaxseed oil diet
OB: Osteoblast
OC: Osteoclast
osterix: Sp7 transcription factor
P1NP: Procollagen type I N-terminal propeptide
PA: Palmitic acid; β-catenin: catenin beta1
RT-qPCR: Real-time quantitative polymerase chain reaction
RUNX2: Runt-related transcription factor 2
WB: Western blot
β-actin: Actin, beta
ω-3 FA: Omega-3 unsaturated fatty acid
